# Nonsuppressible viremia during HIV-1 therapy meets molecular virology

**DOI:** 10.1172/JCI167925

**Published:** 2023-03-15

**Authors:** Ann Emery, Sarah B. Joseph, Ronald Swanstrom

**Affiliations:** 1 Lineberger Comprehensive Cancer Center,; 2 Department of Microbiology and Immunology,; 3 UNC HIV Cure Center, and; 4 Department of Biochemistry and Biophysics, University of North Carolina at Chapel Hill, Chapel Hill, North Carolina, USA.

## Abstract

HIV-1 replication can be suppressed with antiretroviral therapy (ART), but individuals who stop taking ART soon become viremic again. Some people experience extended times of detectable viremia despite optimal adherence to ART. In this issue of the *JCI*, White, Wu, and coauthors elucidate a source of nonsuppressible viremia (NSV) in treatment-adherent patients — clonally expanded T cells harboring HIV-1 proviruses with small deletions or mutations in the 5′-leader, the UTR that includes the major splice donor site of viral RNA. These mutations altered viral RNA-splicing efficiency and RNA dimerization and packaging, yet still allowed production of detectable levels of noninfectious virus particles. These particles lacked the HIV-1 Env surface protein required for cell entry and failed to form the mature capsid cone required for infectivity. These studies improve our understanding of NSV and the regulation of viral functions in the 5′-leader with implications for rationalized care in individuals with NSV.

## Low level viremia on therapy

People living with HIV-1 (PLWH) who are not on therapy have virus particles in their blood that can be detected using a PCR assay, which typically shows levels of 5,000–100,000 viral RNA copies per ml. Suppressive antiretroviral therapy (ART) reduces the amount of viral RNA in the blood to below the limit of detection with clinical tests, i.e., less than 20 or 40 copies/ml, depending on the test. Despite the use of potent ART, the vast majority of PLWH still have a low level of viremia (LLV) that remains below the limit of detection of the clinical test, but can often be detected by highly sensitive assays (1, 2). Multiple studies have shown that ART intensification does not reduce LLV (3, 4) despite continued sensitivity of the virus to the drug regimen (3), suggesting LLV has a source other than viral replication. A surprising feature of LLV is its composition, in which groups of identical sequences (i.e., clones) can persist over time (5–7). Further, LLV can contain replication-competent variants (5, 7, 8) along with defective viral genomes still capable of making particles (9). While LLV is dominated by clonal RNA sequences, proviral genomes in circulating CD4^+^ T cells are dominated by different HIV-1 clones, i.e., it is often hard to find the cells responsible for producing the LLV (5, 6). Our current view of LLV holds that many infected T cells in the latent viral reservoir exist, with only a few capable of producing viral particles. Subsets of these T cells can clonally expand and produce enough viral particles to generate LLV, but still represent only a small fraction of the latently infected T cells. Virus expression and production without viral replication and the expansion and contraction of infected T cell clones all occur in the background of suppressive ART, informing us about the nature of the persistent latent viral reservoir that necessitates lifelong treatment.

In this issue of the *JCI*, White, Wu, and coauthors (10) report on their analysis of nonsuppressible viremia (NSV) in four people on successful ART. The report describes the group of people with persistent viremia above 40 copies/ml that cannot be suppressed with therapy intensification or optimization, which defines NSV. An earlier study by Elvstam et al. found that over a 5.7-year period, approximately 9% of patients on ART experienced elevated detectable viremia (11), although qualifying viral loads included many people with episodic elevated measurements without persistent NSV. Similarly to studies of LLV, viral sequencing and integration site analyses have revealed that NSV is dominated by clonal viral RNA. In some cases, HIV-infected cells producing NSV contain genetically identical expanded clones that can include infectious virus (12, 13).

The paper by White, Wu, and coauthors (10) starts out noting the confusion NSV causes in patient care when persistent viremia appears in someone on ART, then goes on to characterize the virus in four people in care with NSV. Remarkably, the viral populations in all four people contained mutations in the 5′-leader, also known as the 5′ UTR. These mutations inactivated the major splice donor (MSD) site, while still allowing the production of noninfectious virus particles from clonally expanded T cells.

## HIV-1 replication requires the 5′-leader

**Figure 1:**
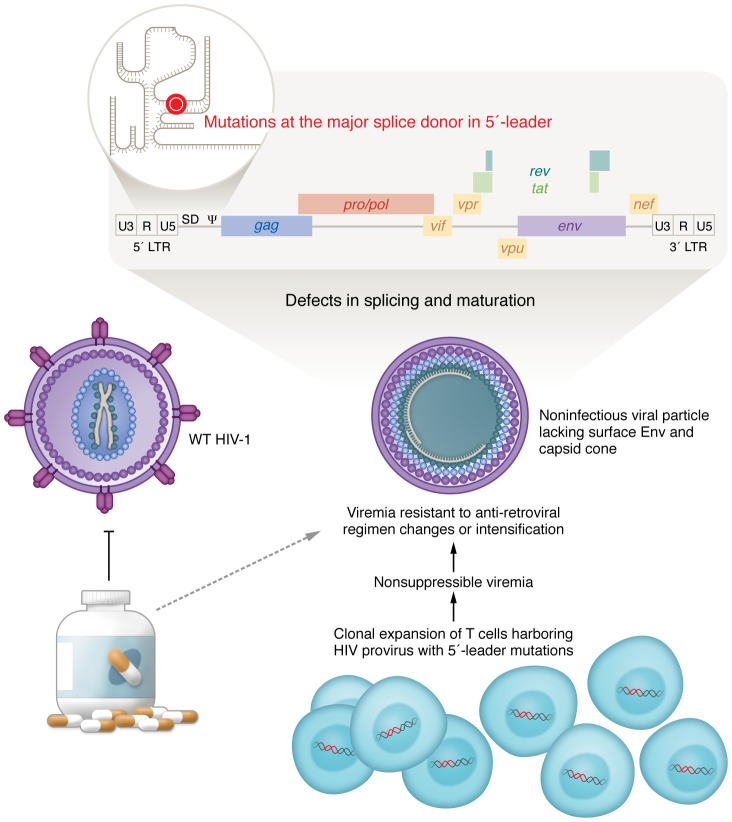
HIV-1 provirus mutations in the 5′-leader underlie the molecular basis of noninfectious NSV. Clonal expansion of T cells expressing an HIV-1 provirus with a mutation in the 5′-leader can result in detectable virus in spite of adequate therapy. The viruses produced have mutations at the MSD site in the 5′-leader of the RNA genome where there are a number of structural and sequence-based functional determinants for viral replication. These viral particles are distinct from wild-type HIV-1 in that they lack detectable surface Env protein and have failed to undergo maturation to produce the mature capsid cone.

The 5′-leader of the full-length HIV-1 transcript is about 790 nucleotides of the 9,800 nucleotide RNA genome and contains numerous structural and sequence-based control elements required for viral replication (Figure 1). Efficient RNA transcription from the viral promoter requires binding of the viral protein Tat to the RNA TAR loop (14). A dimerization sequence (DIS) initiates the base-pairing interaction between two HIV-1 transcripts, resulting in the packaging of two copies of viral RNA in each virion. Packaging of the dimerized genomes into the Gag protein lattice that assembles at the cell membrane is facilitated by the RNA packaging signal (Psi/Ψ) (15). The primer binding site binds to a cellular transfer RNA (tRNA) that is required to prime/initiate viral DNA synthesis (reverse transcription). Another key element of the 5′-leader is the MSD site that initiates all downstream splicing (16).

The 5′-leader can fold into multiple secondary structure conformations that determine whether the viral RNA transcript remains as a full-length viral RNA or is spliced and translated as a subgenomic mRNA (17). Secondary structure also determines whether the MSD site is exposed or secluded and may dictate the balance between unspliced and spliced transcripts (18). Unspliced full-length transcripts can be packaged as dimeric genomic RNA or serve as functional mRNA for translation into the viral Gag and Gag-Pro-Pol polyproteins. Splicing from the MSD site is required to generate the mRNAs for all other viral proteins.

## Proviruses with defective 5′-leaders make noninfectious virus particles

When White, Wu, and coauthors (10) looked at the viral genomes present in four cases of NSV, they found mutations and small deletions that removed or incapacitated the MSD site and that, given the tightly spaced other regulatory sequences, could reasonably impact other 5′-leader control elements. These viruses represented clones, indicating that clonally expanded T cells produced the defective virus particles. Surprisingly, the same specific deletion spanning the MSD site was present in three of the participants, with a point mutation in the MSD site present in the fourth participant. These mutations predictably reduced splicing of viral transcripts, and yet enough spliced transcripts were produced, via suboptimal nearby alternative MSD sites, to yield noninfectious virus particles lacking the surface Env protein. During budding and virion release, wild-type particles cleave the Gag protein to form the capsid cone. However, the mutant virus particles were missing this mature cone morphology and resembled immature particles normally associated with unprocessed Gag protein. This combined loss of the surface Env protein and the absence of the mature capsid cone easily accounts for particle noninfectivity. The authors showed that other regulatory functions in the 5′-leader continued, albeit at reduced efficiencies. Each of the patients with NSV had at least one of these defective proviruses that was expressed to produce particles, while one person (participant 2 [P2]) had an NSV population composed of separate viral lineages with two similar but distinct 5′-leader deletions, an intact viral lineage with a mutation in the MSD site, and also a fully infectious virus.

## Implications

We now know that it is possible for proviruses to produce virus particles with viral RNA even when the MSD site is gone. There are reported examples where the virus particles in elevated NSV are infectious, although not replicating given the presence of antiviral drugs (12, 13). White, Wu, et al. now provide examples of defective viral genomes producing particles at sufficient levels to require investigation (10).

The authors estimate that NSV occurs at a frequency of about 1 in 250 cases of people on suppressive therapy (10). Sequencing of the 5′-leader of virus in people with NSV will help round out our understanding of the mechanisms of NSV and may provide the impetus to follow more cases to get a precise estimate of its frequency.

Several mysteries remain about how these defective particles are made. First, it is unclear what is happening with splicing when the MSD site is missing and why transcripts that initiate splicing are skewed toward complete splicing, causing a reduction in the partially spliced Env mRNA. Second, why are the particles in the immature form? The viral Gag-Pro-Pol polyprotein precursor is made as an alternative translation product of Gag translation and thus independent of splicing. One would assume that if Gag is made, then Gag-Pro-Pol is also produced. Importantly, the Pro in Gag-Pro-Pol is the viral protease tasked with cleaving the Gag and Gag-Pro-Pol polyprotein precursors. White, Wu, and coauthors (10) showed that Gag was cleaved, which would be expected if Gag-Pro-Pol was made and assembled into the virion. However, the question of why the virion exists in an immature conformation remains unanswered. The findings imply that these particles lack a component, resulting in a failure of some heretofore-unknown nucleation event needed to initiate capsid cone formation.

What limits the rate for generating NSV? Viral DNA synthesis makes one mistake per three proviruses generated (19). The viral polymerase is error prone in making point mutations, and the generation of deletions is mechanistically related to recombination for retroviruses (20, 21). Given the large population of replicating virus, many mutations are generated daily, and the types of mutations identified in White, Wu, et al. are clearly incorporated into the latent reservoir and have been seen in LLV (22), indicating that the generation of such genomes is unlikely to limit viral production. Sufficient clonal expansion of a cell expressing this type of virus can clearly result in substantial viremia despite the presence of a defective genome. This possibility is consistent with observations from one of the patients in which the T cell clone with the NSV defective virus represented half of all cells containing viral DNA in the blood. However, in other cases, the expanded and expressing T cell clone giving rise to NSV was a minor component of the reservoir, and the size of these infected clones poorly correlated with levels of NSV. A driver of T cell clonal expansion may be the antigen specificity of the cell and frequent exposure to that antigen. Finally, the defective viral genome must have integrated into a site that allows expression in the context of the host cell chromatin. Some, and likely all, of these factors must happen to give rise to NSV.

It is also possible that the relatively infrequent detection of persistent NSV only occurs in people with inefficient immune surveillance of virus-producing cells that would normally limit the size of such a clone. This would be the opposite outcome of what is seen in elite controllers who can control viremia even in the absence of ART. Recent analysis (23) of viral genomes in elite controllers has shown that cells with intact viral genomes are under strong immune surveillance against expression, leaving only those proviruses that are in a state called “deep latency.” There are two points to consider regarding the possibility that people who experience this elevated NSV struggle to clear cells that are expressing viral proteins, allowing clonal superexpansion, expression of viral proteins, and particle production. First, mutations in the MSD site lead to reduced Env protein expression, both on the virus particle and on the cell surface. If immune surveillance depends on antibodies binding cell-surface Env protein (antibody-dependent cell killing), then these cells have a fortuitous escape mechanism mediated by the virus. The second possibility comes from the analysis of NSV in P2, who had a surprisingly complex NSV population: there were two populations of virus with different 5′-leader deletions, a population with an MSD site mutation, and wild-type virus. This pattern suggests that these viral genomes are capable of expressing as part of multiple expanded cellular clones. P2 also had a larger latent reservoir size, but it is unclear whether the larger reservoir created more opportunity to sample these types of virus-producing cells or whether an inability to efficiently clear virus-expressing cells resulted in a larger steady-state reservoir through more extensive clonal expansion. In this model, ART blocks viral replication, but the host immune selection struggles to clear virus-expressing cells. The opposite pattern occurs in elite controllers, where immune selection efficiently removes cells expressing virus (23). It will be important to understand whether there is an underlying reason why these NSV patients harbor expanded clones expressing viral proteins (10). If it is due to poor immunologic control of virus-expressing cells, this would emphasize the need to maintain potent regimens and possibly avoid drug-reducing regimens.

## Version 1 03/15/2023

Electronic publication
